# Nurses' sense of organizational support, Self‐esteem and perceived professional benefits: A mediating model

**DOI:** 10.1002/nop2.1457

**Published:** 2022-12-09

**Authors:** Mingjing Wang, Limin Wang, Caihong Lu

**Affiliations:** ^1^ School of Nursing Tongji Medical College of Huazhong University of Science and Technology Wuhan China; ^2^ Wuhan Union Hospital Wuhan China

**Keywords:** nurses, perceived organization support, perceived professional benefits, self‐esteem, structural equation modelling

## Abstract

**Aim:**

To explore the mediating role of self‐esteem in the relationship between perceived organizational support and professional benefits among Registered Nurses in China.

**Design:**

This was an online, cross‐sectional study involving 1850 nurses from six hospitals in China.

**Methods:**

Data were collected using a 4‐part questionnaire including a sociodemographic questionnaire, perceived organizational support scale, self‐esteem scale and brief nurses' perceived professional benefits questionnaire from September to November 2021. Structural equation modelling (SEM) was used to explore the associations among them.

**Results:**

Perceived organizational support was positively correlated with self‐esteem and perceived professional benefits among nurses, whereas self‐esteem positively predicted nurses' perceived professional benefits . Self‐esteem partially mediated the relationship between the two variables. The ratio of the mediating effect to the total effect was 16.7%.

## 1 INTRODUCTION

As professional helpers, nurses are in a high‐risk and high‐pressure working environment and often experience traumatic events or scenes, such as patient death; therefore, they are more probably to suffer trauma and lead to job burnout. Burnout is a psychological syndrome that is characterized by emotional exhaustion, depersonalisation and low personal accomplishment. It has been proven to be closely linked to the intention to leave (Dall'Ora et al., 2020). A meta‐analysis showed that the overall prevalence of burnout symptoms among global nurses was 11.23% before COVID‐19 (Woo et al., 2020). Furthermore, the COVID‐19 pandemic has had an even larger impact on nurses' mental health. According to the study by Galanis (Galanis et al., 2021), during the COVID‐19 outbreak, 34.1%, 15.2% and 12.6% of nurses experienced high levels of emotional exhaustion, low personal accomplishment and depersonalisation, respectively. Therefore, measures should be urgently taken to reduce nurses' job burnout and hinder wastage of the labour force.

Based on the broaden‐and‐build theory of positive emotions, positive emotions such as joy, interest, contentment and love can broaden an individual's momentary thought‐action repertoire and build more lasting personal resources to help people generate more positive emotions and develop better. Continuing to form a virtuous circle, for example, the happiness gained through interaction with others or society, is conducive to personal growth and development, thus bringing more positive emotions (Fredrickson, 2004). Nurses' perceived professional benefits can also be regarded as a positive emotional experience for nurses at work, and maintaining positive work emotions is conducive to promoting nurses' physical and mental health and reducing negative emotions, such as burnout. Therefore, this concept may help solve the problem of burnout among nurses.

## 2 BACKGROUND

The concept of NPPB was developed from benefit finding. Nurses perceive the gains and benefits of the profession in the employment process, and they agree that engaging in the nursing profession can promote their all‐around growth and development. It consists of five dimensions: approval from family, relatives and friends; recognition from team members; positive professional perception; pleasant nurse–patient relationships; and personal growth (Hu et al., 2020). In a previous study, NPPB scores were in the upper‐middle level (Zhan et al., 2020). Several studies have reported that NPPB can reduce job burnout (Hu et al., 2020) and enhance nurses' willingness to stay on duty (Liu et al., 2021), which is of great significance for nurses' mental health and retention of nursing staff. This shows nurses' positive cognitive evaluation of their occupation and can be conducive to individual career development as an internal personal motivating factor.

Perceived organizational support (POS) is an employee's perception of and belief in how the organization values their contributions and cares about their well‐being (Eisenberger et al., 1986). According to organizational support theory based on social exchange, if employees feel the appreciation and support of the organization, they will perform highly at work (Battistelli et al., 2016). POS, including material or emotional support, can influence nurses' organizational behaviour such as absenteeism (Eisenberger et al., 1986), turnover intention (Cakal et al., 2021), in‐role performance and prosocial behaviour (Battistelli et al., 2016). It can also effectively alleviate individual negative emotions, such as burnout (Lowe et al., 2020), depression (Chatzittofis et al., 2021) and is related to psychological well‐being (Caesens et al., 2020). Earlier studies have shown that POS, as a source of social support, can improve nurses' sense of professional benefits (Cheng et al., 2020; Zhan et al., 2020).

Self‐esteem is an important psychological variable that reflects an individual's overall evaluation of self‐worth, competence and suitability (Liu et al., 2019). It is associated with mental and physical health (Kupcewicz & Jóźwik, 2020a). Previous studies have revealed that low self‐esteem is related to higher levels of depression (Sowislo & Orth, 2013), burnout (Sturm & Dellert, 2016) and psychological distress (Duran et al., 2021). Self‐esteem has also been confirmed to have a positive influence on subjective well‐being (Yu et al., 2019), work satisfaction (Feng et al., 2018; Sturm & Dellert, 2016) and quality of life (Kupcewicz & Jóźwik, 2020a). Additionally, the mediating role of self‐esteem has often been explored in studies with subjective well‐being and life satisfaction. A previous study showed that perceived organizational support can help people improve their subjective well‐being and life satisfaction by increasing their self‐esteem (Yu et al., 2019). According to sociometer theory, self‐esteem is an intrinsic reflection of personal interpersonal relationships. If people are admitted and liked by others, their self‐esteem rises, and positive emotional experiences are brought about. If not, it leads to a decline in self‐esteem and results in negative emotions. Thus, psychological well‐being is not improved by self‐esteem but by the individual's perception of the acceptance and attention of others (Leary et al., 1995). This further explains why social support, including organizational support, can enhance individual self‐esteem. Although we have already known that POS can improve NPPB and self‐esteem, few studies explore whether self‐esteem could be a moderator in the relationship between them.

Hence, this study aimed to investigate the status of POS, self‐esteem and NPPB among Chinese nurses and further explore the relationships among them. The research questions were as follows: (a) What is the level of POS, self‐esteem and NPPB among nurses in Wuhan, Hubei Province, China? (b) What is the relationship between POS, self‐esteem and NPPB? (c) Does self‐esteem mediate the relationship between POS and NPPB?

The hypotheses were as follows:

- POS can positively predict nurses' self‐esteem and NPPB.
- Nurses' self‐esteem can be positively related to NPPB.
- Self‐esteem can partially mediate the relationship between POS and NPPB.

## 3 DESIGN

This online cross‐sectional study was conducted from September to November, 2021. The STROBE Checklist was used in this study.

## 4 METHODS

### 4.1 Participants and sampling

Before conducting the formal survey, the research group discussed and developed a preliminary inventory for the pilot study. In total, 163 nurses from different clinical departments were selected using cluster sampling. Based on the results of the preliminary investigation, a formal questionnaire was administered. The sample size was calculated using the formula *N* = 4 (Uα S/δ)^2^. The standard deviation (S) and tolerance error (δ) obtained by the pre‐survey were 10.85 and 1.67, respectively. The alpha (α) was 0.05 for two‐tailed tests. The required number was 648 after calculation, and the final sample should be expanded by 10%–20%, considering the invalid questionnaire. Therefore, the required sample size for this study was between 713 and 778.

The formal study was conducted in six class A tertiary hospitals in Wuhan, Hubei Province, China. Participants were recruited using a multistage sampling method. Initially, a stratified sampling method according to the type of hospital (general hospital or specialized hospital) and the number of nurses was used to choose the investigated hospital. The 15 general hospitals were divided into three floors according to the number of nurses less than 1,000, 1,000–2000 and more than 2000. Ten specialized hospitals were divided into two floors according to the number of nurses: less than 500 and more than 500. We randomly selected hospitals on each floor. At this stage, the two military hospitals were exclusive. Then, we purposely and conveniently selected the departments in each hospital. Because of the statistically significant differences in daily work, nurses in sterilization supply rooms and other auxiliary departments were excluded. Finally, we contacted nursing managers and sent a web link (https://www.wjx.cn) to investigate nurses in each department. At this stage, Registered Nurses who were not interns voluntarily participated in the study. Nurses who were absent due to vacation, advanced study, etc. were excluded.

A total of 2,586 nurses were invited to participate in this study. After excluding invalid questionnaires, we collected nurses who had worked for more than 2 years for secondary analysis. A total of 1850 nurses were eventually included in this study.

### 4.2 Measures

#### 4.2.1 General information

Self‐designed general information and demographic questionnaires including gender, age, hospital, department, position, working experience, professional title, monthly income, educational level, marital status and night shift, were asked to fill in.

#### 4.2.2 Perceived organizational support

The Nurses' Perceived Organizational Support Scale (SPOS), which was compiled by Eisenberger (Eisenberger et al., 1986) and revised by Zuo and Yang (2009), was adopted and has been proven to have good reliability and validity among nurses (Yu et al., 2019). It contains 13 items and two dimensions: emotional support (items 1–10) and instrumental support (items 11–13) and uses a 5‐point scoring method, 1 = very non‐conforming and 5 = very conforming. The higher the score, the better the sense of organizational support. In this study, the Cronbach's α of the scale was .979, and the two dimensions were 0.977 and 0.929, respectively.

#### 4.2.3 Self‐esteem

The self‐esteem scale (RSES) was originally compiled by Rosenberg in 1965 (Rosenberg, 1965). It measures individuals' overall self‐esteem and has good reliability and validity (Sang et al., 2017). The Chinese version of the RSES was used in this study and has been widely used by Chinese people (Sang et al., 2017; Shi et al., 2015; Yu et al., 2019). It contained 10 questions and one dimension. The items are simple, clear, easy to measure and scored. Using a 4‐point scoring method, scores were 1–4 (1 = very inconsistent, 4 = completely consistent), and the scale's total score was 10–40. Higher scores indicate higher self‐esteem. Item 8 was positively scored according to Chinese culture (Wu et al., 2017). In this study, the Cronbach's α for the RSES was 0.874.

#### 4.2.4 Perceived professional benefits

The Chinese Perceived Professional Benefits Questionnaire was developed by Hu and Liu et al. in 2013 (Hu & Liu, 2013) and simplified by Hu et al. (2020). The brief nurses' perceived professional benefit questionnaire (NPPBQ) was used to measure nurses' perceived professional benefits. It consists of 17 items representing five subscales: positive occupational perception (three items), good nurse–patient relationship (four items), recognition from family, relatives and friends (three items), sense of belonging to a team (three items) and self‐growth (four items). Responses are recorded on a 5‐point Likert scale ranging from 1 (totally disagree) to 5 (very agree). The higher the score, the higher the level of nurses' perceived professional benefits. In this study, Cronbach's α of the NPPBQ was .957, and all subscales were between 0.820 and 0.877.

### 4.3 Analysis

Statistical analysis was performed using SPSS 24.0, in which counting data were described by frequency and percentage. The measurement data were said as means and standard deviations. Group differences in POS, self‐esteem and NPPB scores were tested using *t* tests and ANOVA. Pearson's correlation coefficient was used to analyse the correlations among POS, self‐esteem and NPPB. Hierarchical multiple regression was applied to further detect associations among the variables. In step 1, the latent control variables were added. The two aspects of POS were added in Step 2. Self‐esteem was entered into Step 3. AMOS 26.0 was used to establish a structural equation model and test the mediating role of self‐esteem on the relationship between POS and NPPB. It was considered statistically significant (*p <* .05).

### 4.4 Ethics

This study was approved by theinstitutional research ethics committee of Tongji Medical College of HUST in China. The nurse executives' permission from the selected hospitals and departments was obtained before the investigation. The first page of the questionnaire was linked to the informed consent, and the nurses should click “agree” before answering questions. The questionnaires were filled anonymously. All the information was only visible to the team members, and the exported data were encrypted and saved.

## 5 RESULTS

### 5.1 Demographic characteristics

Among the 1850 participants, 140 were male (7.6%) and 1710 were female (92.4%); the mean age was 32.23 years (*SD* = 5.99), ranging from 23 to 59 years. There were 1,421 participants from general hospitals (76.8%) and 429 participants from specialized hospitals (23.2%). Only 5.8% of the nurses served as nursing managers. The number of participants who worked for 3–5, 6–10, 11–20 and ≥ 21 years was 373 (20.2%), 791 (42.8%), 550 (29.7%) and 136 (7.4%), respectively. The professional titles of the participants were as follows: junior nurses (1,068, 57.7%), middle nurses (734, 39.7%) and senior nurses (48, 2.6%). Detailed characteristics of the participants are presented in Table 1. As shown in Table 1, nurses with different positions, work experience, professional title, monthly income, marital status and night shifts had a statistically significant difference in NPPB.

**TABLE 1:** Demographic characteristics (n = 1850)

Variables	*N* (%)	POS	Self‐esteem	NPPB
		Mean (*SD*)	Mean (*SD*)	Mean (*SD*)
Gender		*t* = −1.640	*t* = −2.082*	*t* = −0.555
Male	140 (7.6)	46.26 (10.97)	30.36 (3.98)	66.32 (11.1)
Female	1710 (92.4)	47.75 (10.30)	31.12 (4.12)	66.82 (10.19)
Hospital		*t* = −0.348	*t* = −1.440	*t* = −1.100
General hospital	1,421 (76.8)	47.59 (10.58)	30.98 (4.11)	66.64 (10.41)
Specialized hospital	429 (23.2)	47.78 (9.58)	31.31 (4.12)	67.26 (9.73)
Position		*t* = −5.970**	*t* = −5.111**	*t* = −8.175**
Nurse	1743 (94.2%)	47.28 (10.31)	30.94 (4.10)	66.31 (10.09)
Nurse manager	107 (5.8%)	53.38 (9.30)	33.02 (3.80)	74.51 (9.84)
Working experience (years)		*F* = 3.599*	*F* = 9.308**	*F* = 10.640**
3–5	373 (20.2)	48.54 (9.44)	30.88 (4.03)	66.97 (9.89)
6–10	791 (42.8)	46.74 (10.50)	30.64 (4.14)	65.45 (10.28)
11–20	550 (29.7)	48.16 (10.72)	31.47 (4.11)	67.83 (10.34)
≥21	136 (7.4)	48.29 (10.13)	32.34 (3.78)	69.85 (9.61)
Professional title		*F* = 2.991	*F* = 16.039**	*F* = 12.624**
Junior	1,068 (57.7)	47.25 (10.10)	30.67 (4.03)	65.92 (9.98)
Middle	734 (39.7)	48.01 (10.75)	31.48 (4.18)	67.71 (10.60)
Senior	48 (2.6)	50.46 (9.35)	33.31 (3.72)	71.79 (8.30)
Monthly income CNY(USD$)		*F* = 9.608**	*F* = 11.294**	*F* = 9.375**
<2000 (<299)	6 (0.3)	40.50 (11.57)	33.17 (5.85)	64.50 (11.66)
2000–4,999 (299–747)	255 (13.8)	45.67 (11.45)	30.16 (4.06)	65.04 (11.06)
5,000–9,999 (748–1,494)	1,344 (72.6)	47.55 (10.04)	31.01 (4.05)	66.60 (9.91)
≥10,000 (≥1,495)	245 (13.2)	50.31 (10.28)	32.22 (4.19)	69.69 (10.69)
Education		*F* = 0.694	*F* = 1.230	*F* = 0.050
Junior college or below	176 (9.5)	48.51 (10.10)	30.64 (4.34)	66.98 (10.21)
Undergraduate	1,661 (89.8)	47.54 (10.39)	31.11 (4.09)	66.77 (10.25)
Postgraduate or above	13 (0.7)	47.54 (9.47)	30.38 (3.33)	66.31 (12.02)
Marital status		*F* = 1.179	*F* = 5.967**	*F* = 12.071**
Single	508 (27.5)	47.23 (9.84)	30.53 (4.15)	64.92 (10.26)
Married	1,303 (70.4)	47.85 (10.56)	31.26 (4.07)	67.53 (10.17)
Others (divorced or separated)	39 (2.1)	45.95 (10.01)	31.44 (4.52)	66.13 (10.30)
Night shifts		*t* = 2.928**	*t* = 4.846**	*t* = 6.279**
No	496 (26.8)	48.80 (10.64)	31.82 (4.00)	69.23 (10.31)
Yes	1,354 (73.2)	47.21 (10.22)	30.78 (4.12)	65.89 (10.09)

* *p* < .05.

** *p*< .01.

### 5.2 Descriptive statistics

The total scores of POS, self‐esteem and NPPB were 47.64 (*SD* = 10.36), 31.06 (*SD* = 4.11) and 66.78 (*SD* = 10.25), respectively. Comparing the scores of the five dimensions of NPPB, the highest and the lowest dimensions were good nurse–patient relationship 4.09 (*SD* = 0.62) and positive occupational perception 3.69 (*SD* = 0.81). The mean scores of all scales and subscales are shown in Table 2.

**TABLE 2:** Descriptive statistics (*n* = 1850)

Variables	No. of items	Response range	Mean ± SD (total)	Mean ± SD (item)
POS	13	13–65	47.64 ± 10.36	3.66 ± 0.80
Emotional support	10	10–50	36.11 ± 8.27	3.61 ± 0.83
Instrumental support	3	3–15	11.53 ± 2.34	3.84 ± 0.78
Self‐esteem	10	13–40	31.06 ± 4.11	3.11 ± 0.41
NPPB	17	17–85	66.78 ± 10.25	3.93 ± 0.60
Positive occupational perception	3	3–15	11.06 ± 2.44	3.69 ± 0.81
Good nurse–patient relationship	4	4–20	16.36 ± 2.47	4.09 ± 0.62
Recognition from family, relatives and friends	3	3–15	11.29 ± 2.25	3.76 ± 0.75
Sense of belonging to a team	3	3–15	11.92 ± 1.89	3.97 ± 0.63
Self‐growth	4	4–20	16.15 ± 2.39	4.04 ± 0.60

### 5.3 Correlation analysis and hierarchical multiple regression

The normality test showed that all the data were normally distributed. The Pearson's correlation results showed that the correlations among all variables were statistically significant (Table 3). In this study, POS positively correlated with self‐esteem (*r* = .484, *p* < .01). The NPPB was statistically significantly positively correlated with POS (*r* = .780, *p* < .01) and self‐esteem (*r* = .594, *p* < .01). Demographic variables explained 5.6% of the variance in NPPB scores. Two aspects of POS and self‐esteem explained 57.2% and 5.3% of the variance in the NPPB scores, respectively (Table 4).

**TABLE 3:** Correlation analysis (*n* = 1850)

Variable	1	2	3	4	5	6	7	8	9	10
1. POS	1									
2. Emotional support	.993	1								
3. Instrumental support	.912	.859	1							
4. Self‐esteem	.484	.472	.473	1						
5. NPPB	.780	.768	.734	.594	1					
6. Positive occupational perception	.761	.757	.689	.539	.904	1				
7. Good nurse–patient relationship	.683	.668	.661	.532	.908	.780	1			
8. Recognition from family, relatives and friends	.615	.607	.572	.454	.833	.691	.659	1		
9. Sense of belonging to a team	.733	.724	.685	.553	.897	.771	.771	.674	1	
10. Self‐growth	.702	.685	.681	.584	.934	.792	.834	.711	.839	1

*Note*: All correlations are statistically significant (*p <* .01).

**TABLE 4:** Multiple linear regression analysis of NPPB (*n* = 1850)

Variables	NPPB		
	Step 1 (β)	Step 2 (β)	Step 3 (β)
Position	0.040	0.046*	0.046*
Working experience	−0.090*	−0.010	−0.015
Professional title	0.142*	0.025	0.009
Monthly income	0.086*	−0.003	−0.012
Marital status	0.088*	0.072*	0.062*
Night shifts	−0.090*	−0.059*	−0.048*
POS			
Emotional support		0.505*	0.441*
Instrumental support		0.291*	0.223*
Self‐esteem			0.267*
*F*	18.149*	387.792*	435.553*
*R* ^2^	0.056	0.628	0.681
△*R* ^2^	0.056	0.572	0.053

* *p* < .01.

### 5.4 Mediation analyses

Perceived organizational support was considered as the predictive variable, self‐esteem as the mediating variable and NPPB as the outcome variable. Each dimension of the scale was represented as an observation variable. Because the self‐esteem scale had only one dimension, the 10 items were divided into five groups by item parcelling. First, we arranged the items into two rows according to the factorial load from high to low, with five items in each row. We then selected two items in each column and packed them into five variables to use as observation variables of self‐esteem. Whether the hypothetical model was fit can be judged using absolute and incremental fit indices.

**FIGURE 1:**
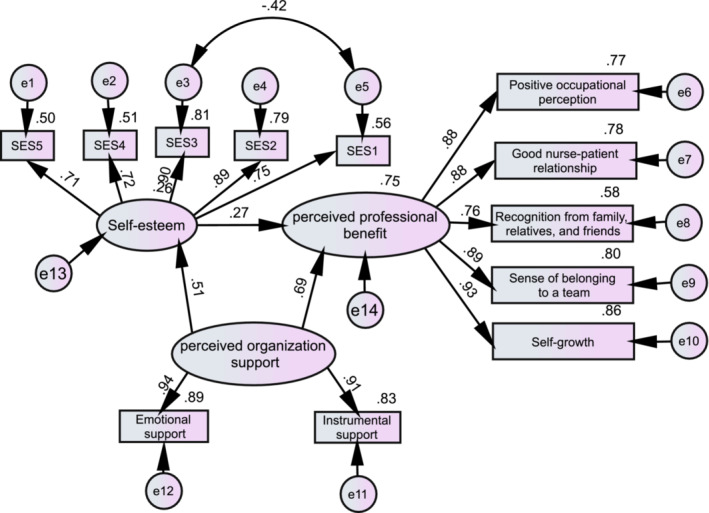
Hypothesized model of POS, self‐esteem and NPPB (standard estimates). CFI, comparative fit index; e, residual error; GFI, goodness of fit index; NFI, normal fit index; RMSEA, root mean square error of approximation.

Since the sample size was larger than 500, the chi‐square values (*χ* ^*2*^) and *χ* ^*2*^ /df ratio were not considered. In our study, the hypothetical model fit indices were good: RMSEA = 0.076, CFI = 0.972, GFI = 0.950 and NFI = 0.969 (RMSEA with an acceptable level of <0.08; CFI, GFI and NFI with an acceptable level of >0.90). The model is illustrated in Figure 1. Path analysis showed that there were direct and indirect paths between POS and NPPB. The direct path showed that POS had a direct positive predictive effect on nurses' perceived professional benefits (β = .69, *p* < .001). The indirect path indicated that POS, by the function of mediating variable “self‐esteem” (β = .51, *p* < .001), positively affected NPPB (β = .27, *p* < .001). The standardized indirect effect was 0.139 (95% CI: 0.116–0.163; *p* < .001). The standardized direct effect was 0.692 (95% CI: 0.657–0.727, *p* < .001), and the standardized total effect was 0.832 (95% CI: 0.810–0.852, *p* < .001). Thus, the ratio of the mediating effect to the total effect was 16.7%. The model indicated that self‐esteem partially mediates the relationship between POS and NPPB.

## 6 DISCUSSION

Our findings showed that POS, self‐esteem and NPPB were above the middle level among the nurses investigated. POS was positively related to NPPB, whereas self‐esteem partially mediated the relationship between the two variables.

The NPPB was 66.78 (*SD* = 10.25), lower than that in the previous study by Hu et al. (2020). This may be due to differences in sampling methods, environments or the impacts of COVID‐19. Because of the lack of studies using the NPPBQ as a research tool, more comparisons were not possible. Our study revealed that nurses can perceive the gains and benefits of their profession and recognize the work value of nursing itself. In all dimensions, the subscale of good nurse–patient relationship scored the highest, which clearly stated that nurses can perceive benefits from a good nurse–patient relationship. It is not difficult to understand why. Nurses' daily work behaviours were highly prosocial. The implementation of prosocial behaviour can bring people happiness, a sense of meaning and efficacy. In particular, recipients of prosocial behaviour express gratitude, which can promote benign interactions as well (Hui et al., 2020). Accordingly, nurses can benefit from the nurse–patient relationships. The score for positive occupational perception was the lowest, indicating that nurses' positive perceptions and recognition of occupation were relatively insufficient. This also meant that NPPB could be further improved, especially in terms of positive occupational perception.

In this study, the total score of POS was 47.64 (*SD* = 10.36), indicating that the sense of organizational support can improve nurses' perception of professional benefits. A high level of POS means that nurses feel that they have obtained valuable resources from the organization, and their contributions have been recognized (Eisenberger et al., 1986). According to the reciprocity principle, nurses may improve their behaviours and build emotional commitment with organizations in return (Al‐Hamdan & Bani, 2021). Moreover, it can further increase nurses' sense of belonging and identity (Tayfur et al., 2021). Thus, POS can undoubtedly promote NPPB. In this study, POS was the most important factor affecting nurses' sense of professional benefits. The score for instrumental support was higher than that for emotional support, which suggests that emotional support was relatively insufficient compared with instrumental support. Therefore, nurse managers should not only pay attention to providing material support to nurses, but also show solicitude for nurses so that personnel can feel support from the organization and improve their sense of organizational support.

The self‐esteem score of nurses was 3.11 (*SD* = 0.41), which was higher than that reported in previous studies among Chinese nurses (Feng et al., 2018; Yu et al., 2019). The main reason may be that, unlike other studies, item 8 was positively calculated according to Chinese culture. In addition, the reliability of the self‐esteem scale can be improved if item 8 is scored positively (Wu et al., 2017). Thus, self‐esteem scores may be higher in this study. Similar to previous studies, our study confirmed that POS can positively predict self‐esteem level (Feng et al., 2018; Liu & Liu, 2016; Yu et al., 2019). Several studies have also pointed out that employees can develop organization‐based self‐esteem (OBSE) referring to the extent to which an individual considers himself capable, important and worthy as a member of the organization; and organizational support statistically significantly affected OBSE, which is consistent with our findings (Chen et al., 2016; Pierce et al., 1989).

Self‐esteem has been proven to be one of the most important variables affecting nurses' professional development (Serafin et al., 2022). The established structural equation model verified the hypothesis that self‐esteem could be a mediator variable between POS and NPPB. This finding indicates that self‐esteem positively predicts NPPB. Furthermore, POS can not only directly affect NPPB, but also indirectly promote nurses' benefit from their careers by improving their self‐esteem. For nurse managers, providing adequate organizational support and enhancing nurses' self‐esteem is important for improving NPPB. Previous research has shown that self‐confidence and interpersonal communication skills training can improve nurses' self‐esteem, interpersonal relationships and communication skills and reduce job burnout (Duran et al., 2021; Shimizu et al., 2003). A study conducted among 212 nurses in Germany found that nurses with an academic education displayed a higher level of self‐esteem. The type of professional training may also influence self‐esteem (Van Eckert et al., 2012). Therefore, training or individual courses aimed at developing self‐esteem are recommended (Kupcewicz & Jóźwik, 2020b). Furthermore, some researchers have suggested that nurse administrators can hire individuals with high self‐esteem as nurses for their ability to fight potential psychological problems (Feng et al., 2018).

### 6.1 Limitations

Although this study investigated the status of POS, self‐esteem and NPPB, and first explored the associations between them, there were still some limitations. First, this study was cross‐sectional; therefore, it can only represent the status of POS, self‐esteem and NPPB at a certain point in time. Second, the nurses included in this study were from the same province in China. Thus, the results cannot be extended to nurses across countries. Future studies could use different methods, such as qualitative research, for further examination.

## 7 CONCLUSION

In summary, our results validated that POS and self‐esteem are important factors that affect NPPB. A sense of organizational support can directly help nurses perceive benefits from their occupation or indirectly help them by boosting their self‐esteem. Nurse managers should take action in both organizational and individual aspects to help nurses find benefits from their careers.

## AUTHOR CONTRIBUTIONS

M.J.W and C.H.L designed the study and collected the data. M.J.W and L.M.W analysed the data. M.J.W wrote the manuscript and revised this article with C.H.L and L.M.W. All authors reviewed and contributed to finalize the manuscript.

All authors have agreed on the final version and meet at least one of the following criteria [recommended by the ICMJE (http://www.icmje.org/recommendations/)]:

- substantial contributions to conception and design, acquisition of data or analysis and interpretation of data;
- drafting the article or revising it critically for important intellectual content.

## FUNDING INFORMATION

This study was supported by the special soft science project for Technological Innovation in Hubei Province, China (2019ADC074) and general projects from the Health Commission of Hubei Province, China (WJ2021M241).

## CONFLICT OF INTEREST

The authors declare that there is no conflict of interest.

## ETHICAL APPROVAL

The study was granted by the Institutional Research Ethics Committee of Tongji Medical College of HUST in China (2021S182).
